# Mucous membrane pemphigoid-associated paronychia with onychomadesis

**DOI:** 10.1186/s12895-019-0083-7

**Published:** 2019-01-23

**Authors:** Salim Alkeraye, Sarah F. Alsukait

**Affiliations:** Dermatology Department, College of Medicine, King Saud University, Riyadh, Saudi Arabia

**Keywords:** Onychomadesis, Mucous membrane pemphigoid, Autoimmune bullous disorders

## Abstract

**Background:**

Mucous membrane pemphigoid (MMP) is an autoimmune blistering disease that is notoriously difficult to treat. Nail involvement in MMP is rare.

**Case presentation:**

We report on a 58 years old man with severe MMP who presented with onychomadesis.

**Conclusion:**

To our knowledge, mucous membrane pemphigoid associated paronychia and onychomadesis have not been reported before. We believe it is important for dermatologists to be aware of this entity.

## Background

Mucous membrane pemphigoid (MMP) is an autoimmune blistering disease that is notoriously difficult to treat. Autoimmune mucocutaneous blistering diseases (AMBD) are characterized by autoantibodies directed against epidermal and basement membrane proteins, leading to blister formation. Pemphigus patients may also present with nail abnormalities, with paronychia and onychomadesis being the most common nail changes observed [1, 2]. Nail lesions in bullous pemphigoid are quite rare. The most frequently associated nail findings were nail loss and ptergyium formation [3, 4]. Only one study reported nail abnormalities in MMP, which described ptergyium formation and atrophy of the finger nails [5]. We here describe an unusual case of onychomadesis in a patient with MMP.

## Case presentation

**Fig. 1:**
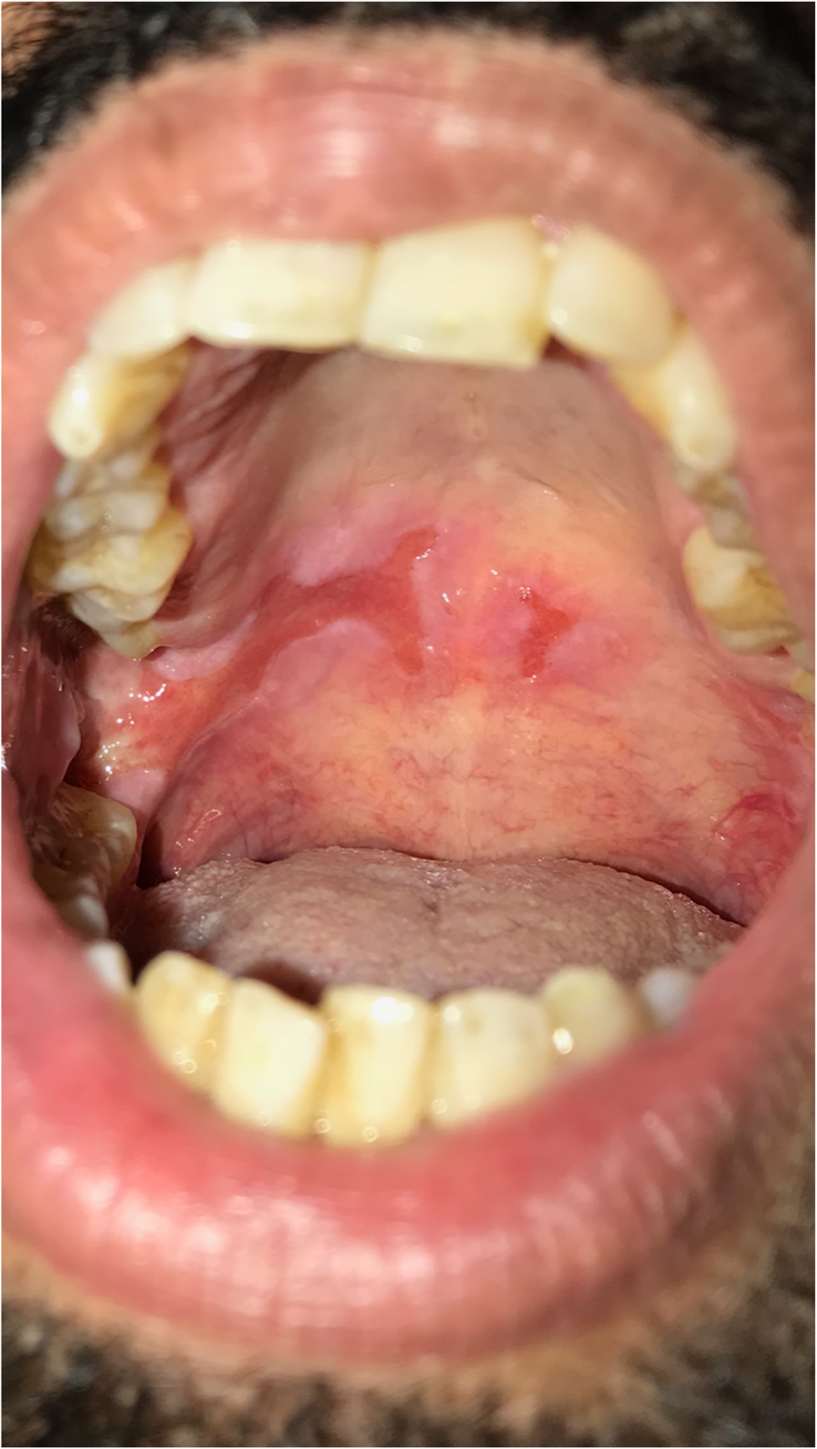
Confluent erosions on the hard and soft palates

**Fig. 2:**
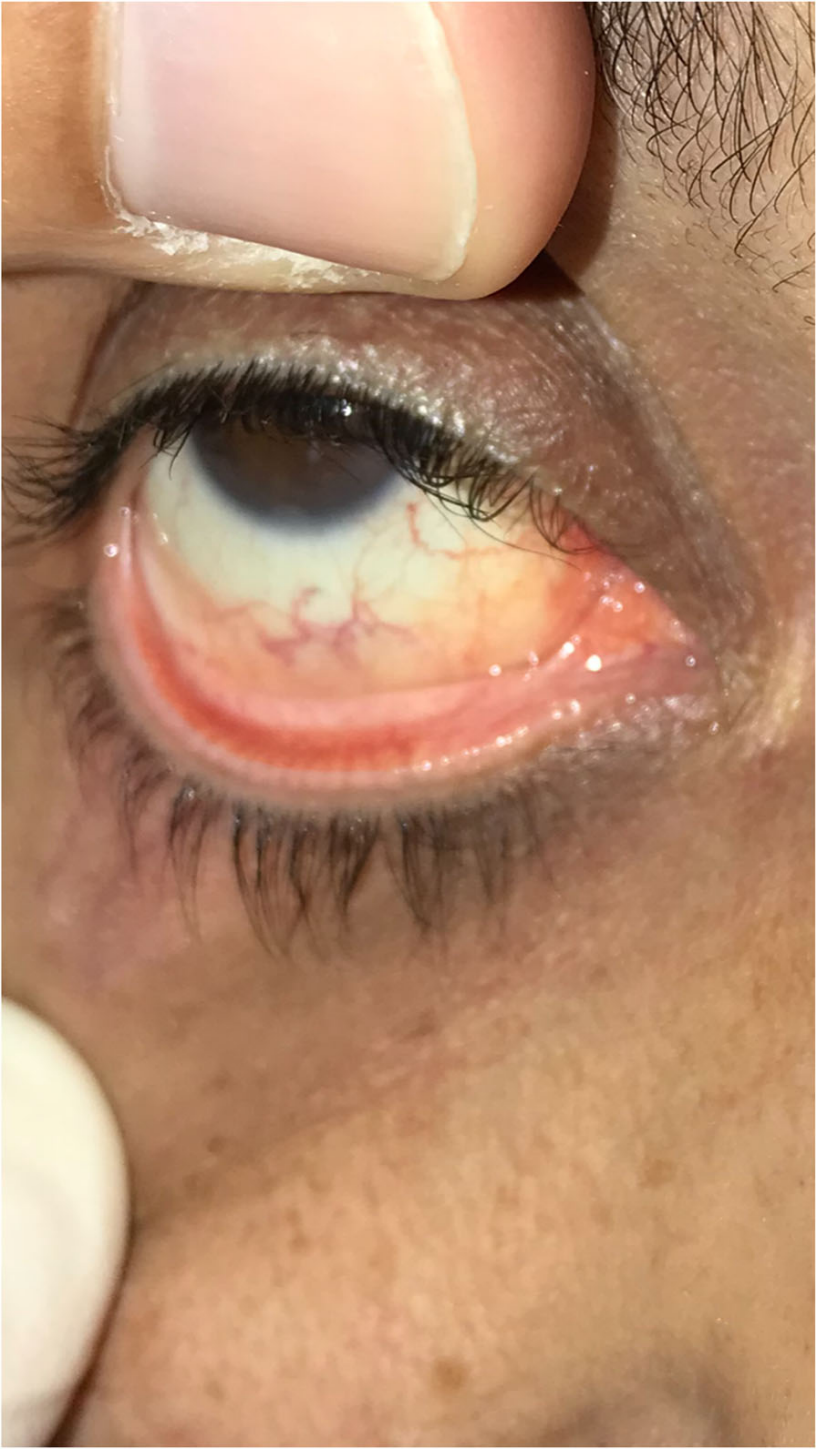
The right eye showing shortening of the fornix

**Fig. 3:**
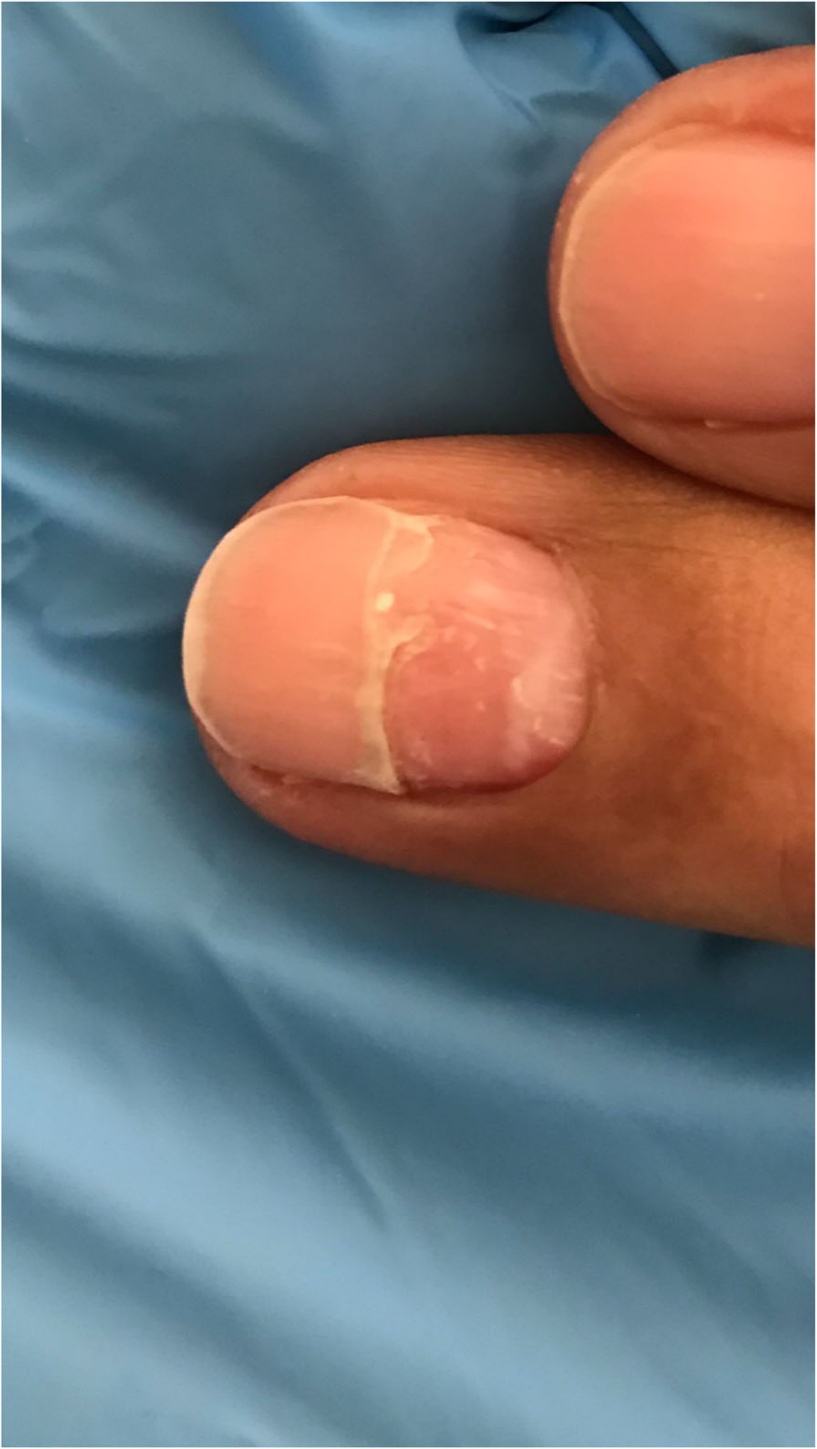
Onychomadesis on the left middle finger’s nail

A 58 years old man presented to dermatology clinic with 2 years history of recurrent painful mouth sores and cutaneous blisters on his extremities and genital area. A review of symptoms was notable for eye irritation, redness and foreign body sensation in both eyes. The patient was not known to have any medical illnesses and was not taking any medications. Physical examination found confluent erosions on the hard and soft palates, buccal mucosa, and on the lateral sides of his tongue (Fig. 1). Skin examination revealed atrophic and hyperpigmented scars on the anterior side of both thighs. We also noticed a small atrophic scar on the penile shaft. His left middle finger showed periungal erythema and swelling that was tender to palpation. Ophthalmologic evaluation revealed chronic conjunctivitis on both eyes with fornix shortening in the right eye (Fig. 2). Nasal scope examination showed few erosions. Laryngoscopy showed erythematous mucosa over the arytenoids. Gastrointestinal evaluation was normal. Histopathological examination of an oral mucosal biopsy showed sub-epithelial blister with underlying chronic inflammation. Immunofluorescence studies were negative. On the basis of the clinical assessment and histopathological results we retained the diagnosis of MMP. The patient was initially treated with 1 mg/kg of prednisone which resulted in a rapid control of his symptoms but when the dose was tapered to 0.5 mg/kg the patient showed signs of disease recurrence. 2 g/kg/cycle of intravenous immunoglobulin therapy IVIG was added. The patient received three cycles on a monthly interval and showed remarkable improvement. Prednisone dose was tapered to 0.25 mg/kg with no signs of disease activity. The left middle finger periungual inflammation had subsided but onychomadesis was noted on the same nail (Fig. 3).

## Discussion and conclusion

The normal human nail immune system is very similar to the hair follicle immune system, including a known area of relative “immune privilege” in the proximal nail matrix, which can constitute a safeguard against autoimmunity [3].

Onychomadesis is characterized by the detachment of the nail plate from the proximal nail fold with persistent attachment to the nail bed and often, but not always, eventual shedding and is due to a severe insult that produces a complete arrest of nail matrix activity [6]. Most commonly, onychomadesis has been reported in association with pemphigus vulgaris and hand–foot–mouth disease, and following chemotherapy or antiepileptic medications [2, 6].

MMP is a chronic autoimmune sub-epithelial blistering disease of the mucous membranes and, less often, the skin. The primary lesion is a vesicle or bulla, and this evolves to become an erosion or ulcer that heals with scarring [7, 8]. Autoantibodies binding to the epithelial basement membrane zone (BMZ) have been demonstrated in this subset, targeting bullous antigens 1 and 2, laminin 332 and laminin 311, type VII collagen, alpha 6 beta 4 integrin, and some nonidentified basal membrane zone antigens [5]. Diagnosis of MMP can be done based on clinical features, histopathological study, and immunopathological (direct and indirect immunofluorescence) and immunochemical studies. Distinction from other subepidermal autoimmune bullous diseases depends on clinical presentation with predominant mucosal involvement [7]. Both lesional skin and mucosal biopsies in our patient demonstrated subepithelial and subepidermal blister formation with underlying mixed inflammatory cell infiltrate, which is consistent with pemphigoid disorders [7]. Direct immunofluorescence (DIF) testing was found to be unexpectedly negative. The diagnosis of MMP depends largely on DIF testing, which is known to be the gold standard [7]. However, many studies conducted on patients with MMP showed DIF sensitivity rates of 70–80% [9–11]. In those studies, MMP diagnosis in patients with negative DIF was formed based on clinical and histopathological features. Sinclair et al. [12] demonstrated that all target antigens found in the normal non-appendageal basement membrane, in specific the epidermal-associated antigens 220-kDa and 180-kDa BP antigens, were expressed by the proximal nail fold, the nail matrix, the nail bed and the hyponychium. Nail abnormalities are rarely involved in pemphigoid disorders. One report described nail dystrophy and ptergyium formation in a patient with MMP [3]. Onychomadesis was also reported in a patient with BP [13].

The paronychia described in our patient was chronologically associated with the disease activity and has cleared after controlling the symptoms, therefore, suggesting a possible association.

In conclusion, we report a case of onychomadesis following an episode of acute paronychia in a patient with MMP. We believe it is important for dermatologists to be aware of this association to avoid additional investigations or treatments.

## Abbreviations

AMBD: Autoimmune mucocutaneous blistering diseases
BMZ: Basement membrane zone
BP: Bullous pemphigoid
IVIg: Intravenous immunoglobulin therapy
MMP: Mucous membrane pemphigoid

## References

[1] Ito T, Ito N, Saathoff M, Stampachiacchiere B, Bettermann A, Bulfone-Paus S (2005). Immunology of the human nail apparatus: the nail matrix is a site of relative immune privilege. J Invest Dermatol.

[2] Habibi M, Mortazavi H, Shadianloo S, Balighi K, Ghodsi SZ, Daneshpazhooh M (2008). Nail changes in pemphigus vulgaris. Int J Dermatol.

[3] Tosti A, Andre M, Murrell DF (2011). Nail involvement in autoimmune bullous disorders. Dermatol Clin.

[4] Gualco F, Cozzani E, Parodi A (2005). Bullous pemphigoid with nail loss. Int J Dermatol.

[5] Burge S, Powell S, Ryan T (1985). Cicatricial pemphigoid with nail dystrophy. Clin Exp Dermatol.

[6] Hardin J, Haber R (2015). Onychomadesis: literature review. Br J Dermatol.

[7] Fleming T, Korman N (2000). Cicatricial pemphigoid. J Am Acad Dermatol.

[8] Ahmed AR, Kurgis BS, Rogers RS 3rd. Cicatricial pemphigoid. J Am Acad Dermatol 1991;24:987–1001. 10.1016/0190-9622(91)70159-y 1869688

[9] Rogers RS, Van Hale HM (1986). Immunopathologic diagnosis of oral mucosal inflammatory diseases. Australas J Dermatol.

[10] Helander SD, Rogers RS (1994). The sensitivity and specificity of direct immunofluoresence testing in disorders of mucous membranes. J Am Acad Dermatol.

[11] Sano SM, Quarracino MC, Aguas SC (2008). Sensitivity of direct immunofluorescence in oral diseases. Study of 125 cases. Med Oral Patol Oral Cir Bucal.

[12] Sinclair R, Wojnarowska F, Leigh I, Dawber RP (2006). The basement membrane zone of the nail. Br J Dermatol.

[13] Benmously-Mlika R, Hammami-Ghorbel H, Mokhtar I (2013). Onychomadesis during bullous pemphigoid. J Am Acad Dermatol.

